# 

**Published:** 1887-10-01

**Authors:** 


### CONTENTS.

**The Oracles of Medicine** - - - - - - 1

**Words of Consolation.**—Chapter LII. The Sufferings of Christ - - - - - - - - 2

**The Doctor's Pulpit.**—Bacteriology - - - - - 3

**Reviews and Notices.**—Consumption: Climatic Treatment - - - - - - - - 4

**Oliver Wendell Holmes.** By Dr. CATERINA - - 5

**Professional Notes.** By a PHYSICIAN - - - 7

**Notes and News.**—Miscellaneous Items.—Trade Societies' Demonstrations.—Hospital Furnishing.—The Preston Infirmary.—Vacancies.—Open-air Concert.—"First Aid to the Wounded" Examinations.—Hospital Finances.—Hospital Extension Scheme in Aberdeen.—The Hospitals Association.—The Foresters' Societies and the Hospitals, etc., etc. - 8

**Annotations.**—Microbes.—A Strange Locum Tenens.—Compulsory Notification of Infectious Diseases - - 11

**The Common Accidents of Every-day Life.** By ROBERT A. P. FORRESTER, B.A. - - - - - 12

**The Matrons' Corner** - - - - - - 12

**East and West.** By LESLIE KEITH - - - - 13

**Sick Nursing in the Sixteenth Century.** By A STUDENT OF MEDIÆVAL INSTITUTIONS - - - - 15

**Recent Drugs and Remedies.**—Electricity - - 16

**Statistics of Alcohol.**—The Consumption of Spirits. By R. R. REDMAYNE - - - - - - - 17

**Cruel Treatment of a Female Patient by her Parents** - - - - - - - 17

**The National Pension Fund** - - - - - 17

**The Editor's Letter-Box** - - - - - 17

**The St. John's Ambulance Carriage** - - - - 17

**Amusements with Prizes, for all Ages.**—For Men and Women.—The Children's Corner - - - - 18

